# 3D Polyaniline Nanofibers Anchored on Carbon Paper for High-Performance and Light-Weight Supercapacitors

**DOI:** 10.3390/polym12112705

**Published:** 2020-11-16

**Authors:** Sami ur Rahman, Philipp Röse, Mit Surati, Anwar ul Haq Ali Shah, Ulrike Krewer, Salma Bilal

**Affiliations:** 1 National Centre of Excellence in Physical Chemistry 1, University of Peshawar, Peshawar 25120, Pakistan; samiurrahman720@gmail.com; 2 Karlsruhe Institute of Technology (KIT), Institute for Applied Materials, 76131 Karlsruhe, Germany; philipp.roese@kit.edu (P.R.); mitsurati@gmail.com (M.S.); 3 Institute of Chemical Science, University of Peshawar, Peshawar 25120, Pakistan; anwarulhaqalishah@uop.edu.pk

**Keywords:** polyaniline, supercapacitors, nanofibers, sodium phytate, activated carbon paper

## Abstract

In the field of advanced energy storage, nanostructured Polyaniline (PANI) based materials hold a special place. Extensive studies have been done on the application of PANI in supercapacitors, however, the structure–property relationship of these materials is still not understood. This paper presents a detailed characterization of the novel sodium phytate doped 3D PANI nanofibers anchored on different types of carbon paper for application in supercapacitors. An excellent relationship between the structures and properties of the synthesized samples was found. Remarkable energy storage characteristics with low values of solution, charge transfer and polarization resistance and a specific capacitance of 1106.9 ± 1.5 F g^−1^ and 779 ± 2.6 F g^−1^ at current density 0.5 and 10 Ag^−1^, respectively, was achieved at optimized conditions. The symmetric supercapacitor assembly showed significant enhancement in both energy density and power density. It delivered an energy density of 95 Wh kg^−1^ at a power of 846 W kg^−1^. At a high-power density of 16.9 kW kg^−1^, the energy density can still be kept at 13 Wh kg^−1^. Cyclic stability was also checked for 1000 cycles at a current density of 10 Ag^−1^ having excellent retention, i.e., 96%.

## 1. Introduction

The main characteristic of modern energy research is the provision of an adequate and sustainable energy supply for all types of technologies and applications. As fossil fuel resources deplete quickly, a replacement is required to meet the needs of the future world. Insufficient resources also force innovations that can use the resource more efficiently. In addition, the need for devices with high mechanical flexibility has increased in recent years. For example, foldable electronic devices, roll-up displays, bendable medical and portable devices have been developed for unusual uses [1,2,3]. This also includes mechanically flexible energy storage devices. Although there are numerous flexible electronic products commercially available today, advances in flexible energy and power sources for these devices have been slow [4,5]. In order to remove this limiting factor, extensive efforts are under development to produce flexible and high-performing energy storage devices [6,7,8]. Supercapacitors offer an effective solution here, as they have great flexibility in their construction as well as good compatibility with other electronic devices and outstanding electrochemical performance [9,10]. They are classically composed of two electrodes (cathode and anode), separator, electrolyte and packing materials [11,12]. The two electrodes are the important parts that allow the devices to be extremely flexible. Conventionally, metal foils (Cu, Au, Al and Ti foils) [13,14,15], and certain plastics [16] are used as substrates for making flexible electrodes. However, these substrates have recognizable disadvantages. The heavy weight of the electrochemically inactive metals adds significantly to the overall weight of the device. Furthermore, they can be eagerly oxidized and cause a substantial drop in electrical conductivity. For the flexible plastic substrates, the weak attraction between active materials and plastic substrates often leads to the detachment of active materials during the electrode distortion induced by cycling, which really limits their durability [13,14,15,16]. Many research groups stated that using self-supported materials including graphene foam and film [17,18,19], carbon nanotubes [20,21,22], electrospun carbon nanofibers [23,24], carbon foam [25], carbon hybrids [18,26] as backbones to assemble freestanding supported hybrids electrodes. Conversely, most self-supporting carbon electrodes are comparatively expensive and complicated to manufacture, which considerably limits the large-scale use of free-standing carbon composites for supercapacitors. Therefore, the development of freestanding supported electrodes with promising mechanical strength and flexibility is in great demand for energy storage devices [27,28].

As one of the oldest flexible products, invented 2000 years ago, paper is still used to record and package information. It is one of the cheap alternatives to conventional flexible substrates [15]. Since the 1960s, carbon-based paper has been researched and used as a substrate for various types of electronics, e.g., paper electronics [29], sensors [30], displays [31], transistors [32], radio frequency identification devices [33], light-emitting diodes [34] and generators [35]. Carbon-based papers are suitable electrode substrates, because of their widespread availability, low cost, light weight, recyclability, flexibility, and environmental friendliness [36,37,38]. Typical paper electrodes are made by depositing or growing active materials on conventional base material backbones. Due to their poor electrical conductivity, however, additional chemical changes (coating of conductive layers) are required to make them more electrically conductive [39,40]. Carbon-based papers in this regard have the advantages of improved capacity and flexibility when used to design supercapacitive electrodes.

While paper electrodes have received a lot of attention in the research community and have shown great prospects in flexible energy storage, electrical conductivity is a serious factor that can severely disrupt the electrochemical performance of simple paper-based electrodes. In addition, most paper-based electrodes are made by mixing active materials with polymeric binders such as polyvinylidene fluoride (PVDF), polytetrafluoroethylene (PTFE), carboxymethyl cellulose (CMC), and conductive additives such as carbon black using suitable solvents [41]. The binders are used to glue the active materials and conductive agents, as well as the current collector, while the conductive agent helps to transfer electrons from active materials to the current collector. The introduction of these isolated binders increases the interfacial resistance between particles as well as the resistance of the electrodes. In addition, the binders and additives make up about 20–40% of the total electrode mass, which is realized as “dead mass” because they do not subsidize charge storage, but rather reduce the electrochemical performance of both the electrodes and, consequently, the devices [42]. In general, a high specific capacity together with long-term stability during the cycle, low cost and light weight of the manufactured device is a major requirement for industrial applications of a material.

To overcome these deficiencies, we investigated in this thesis the application of sodium phytate-doped 3D-PANI nanofibers [43], which are applied to lightweight, low-cost carbon papers for energy storage in supercapacitors. The strategy of binderless cell assembly makes this approach even more attractive. The state of the art shows that PANI is mainly tested on metal surfaces such as steel, gold and nickel, which contributes significantly to the weight of the device. It is a fact that the same PANI material has different specific capacities on different substrates. In general, it shows a high capacity on the surface of gold. In the present study, however, one of the PANI-coated carbon papers doped with sodium phytate shows a specific capacitance value that is higher than that of gold. The material is highly conductive and, under optimized conditions, offers extremely good energy storage properties. These results are very promising and represent a step towards more cost-effective, flexible, and light supercapacitive electrodes.

## 2. Materials and Methods

### 2.1. Materials and Methods

PANI samples were synthesized from commercially available Stock: aniline (C_6_H_5_NH_2_), sodium phytate (C_6_H_17_NaO_24_P_6_), ammonium persulphate (NH_4_)_2_S_2_O_8_, dimethylformamide (DMF, C_3_H_7_NO) were purchased from Sigma Aldrich (St. Louis, MI, USA and Darmstadt, Germany) and sulphuric acid (H_2_SO_4_) was provided by Scharlu (Barcelona, CAT, Spain). Aniline was freshly distilled twice to remove any types of impurities. After distillation, the aniline was kept in a refrigerator for further use. The rest of the chemicals were used as received. Deionized water was utilized for sample synthesis and washing purposes. As coating material, the active carbon papers Sigracet GDL 29AA (ACP-1, without microporous structure), GDL 35BC (ACP-2, microporous structure) from SGL Carbon and Freudenberg H23C2 (ACP-3, microporous structure) were used (see Supplementary Information, Tables S1–S3). Gold foil (99.99%, 0.127 mm thickness) was purchased from ChemPur (Karlsruhe, BW, Germany). 

**Figure 1:**
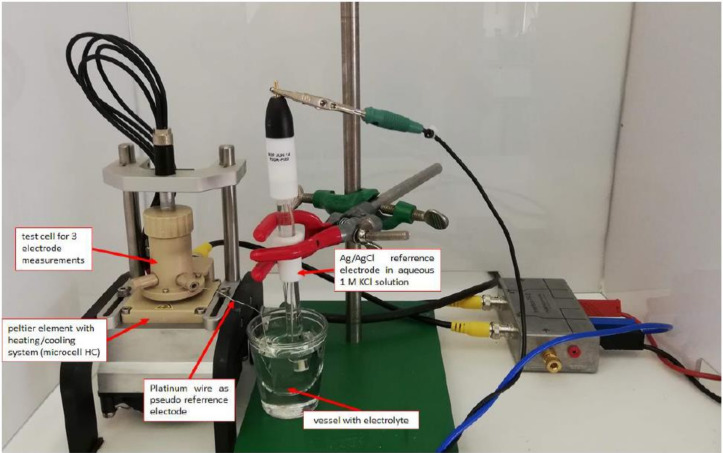
Electrochemical experimental setup with a Microcell HC Cell Stand for closed cells with a TSC Battery (standard) cell from rhd instruments GmbH and Co. KG with an additional Ag/AgCl reference.

Surface morphology and elemental composition analysis of the synthesized PANI samples were done by Helios G4 CX FEI Deutschland GmbH, Berlin, Germany. Imaging of the coated PANI composites was done with a VHX-900F digital microscope from Keyence. Nitrogen absorption–desorption isotherms were measured using Brunauer–Emmett–Teller (BET) method on a Surface Area Analyzer from Quanta Chrome Instrument (Version 11.04, Boynton Beach, FL, USA). Pore volume was obtained from the adsorption branches by using the Barret–Joyner–Halenda (BJH) method. Electrochemical experiments were done using a Microcell HC Cell Stand for closed cells from rhd instruments GmbH and Co. KG (Darmstadt, Germany) (Figure 1). The cell stand contains a Peltier element for active heating and cooling of the cell. As casing, the TSC Battery (standard) was used. It is a closed measuring cell intended for assembling half or full cell batteries or supercapacitors. Both two- and three-electrode measurements can be performed by inserting a reference electrode from the side. The cell contains stainless steel current collectors embedded in PEEK. A good connection between all components of the cell is ensured by a spring that presses upon the current collector with 2.3 Nm. The Microcell HC Cell Stand is connected with a Eurotherm temperature controller and a Gamry Instruments Interface 1010E (Gamry Instruments Framework Software Version 7.8.1, Build 7232, Warminster, PA, USA).

The properties of the PANI-composites and electrodes, i.e., specific capacitances, energy densities, power densities, maximum power density and coulombic efficiency are calculated from the discharge slopes of the galvanostatic charge/discharge (GCD) and cyclovoltammetry (CV) experiments. The Capacitance *C* in Farads (F) is given by the Equations (1) and (2) when calculated from GCD or CV results, respectively:

(1) $$C=\frac{Idt}{dU}$$

(2) C=∫Itdt2ΔU=∫Itdt2ν×ΔU

where *I* is the discharge current in amperes (A), *dU*/*dt* the slope in volts per second (V·s^−1^), *f* the frequency in Hertz (Hz), $\int I\left(t\right)dt$ the integration of the area enclosed by the cyclic voltammetry divided by 2 to account for the discharge part, ν the scan rate in (mV·s^−1^) and Δ*U* the voltage window of the CV curve. To account for the amount of the PANI coating on the composites, the specific gravimetric capacitance *C_m_* in Farads per gram (F·g^−1^) was used (Equation (3)):

(3) $$C_{m}=\frac{2C}{m}$$

where *m* is the mass of the electrode in gram (g) of the active material of one electrode. In the case of a symmetric cell, where both the working and counter electrode are of the same material, size, and mass the specific gravimetric capacitance has to be doubled.

The gravimetric energy and power densities of the composites are calculated from the results of the GCD experiments. Gravimetric energy density *E* in Watt-hours per kilogram (Wh kg^−1^) is calculated using Equation (4):

(4) $$E=\frac{C\Delta U_{max}^{2}}{2m}\times \frac{1000}{3600}$$

where *U_max_* is the maximum voltage in volts (V) and m is the mass of the active material of both electrodes in kilogram (kg).

The gravimetric power density *P* in Watt per kilogram (Wkg^−1^) is calculated by Equation (5).

(5) $$P=\frac{E\times 3600}{\Delta t}$$

The coulombic efficiency η (%) is calculated by Equation (6):

(6) $$\eta =\frac{t_{d}}{t_{c}}\times 100\%$$

where *t_d_* and *t_c_* are the discharge and charge times.

### 2.2. General Procedure for the Synthesis of Sodium Phytate Doped PANI

Solutions with 0.5%, 5%, and 10% (*w*/*v*) sodium phytate were prepared in H_2_O at room temperature. Then 2.5 mL from the respective sodium phytate solution was mixed with 497 μL (5.50 mmol) aniline in 5 mL H_2_O. A second solution containing ammonium persulfate (1.0 mM in H_2_O) was prepared. The solutions were kept in refrigerator at 4 °C for 15 min. Polymerization was performed by adding 1.0 mL of sodium phytate solution to 0.5 mL of ammonium persulfate followed by rapid mixing. After 5 min, the color of the mixture turned dark green indicating the formation of PANI. After 10 min, the mixture was filtered, and the product was washed with acetone and dried under vacuum. The respective PANI samples were labeled as S1 (0.5%), S2 (5%) and S3 (10%), according to the amount of dopant.

### 2.3. Preparation of the Electrode Composite Materials

**Figure 2:**
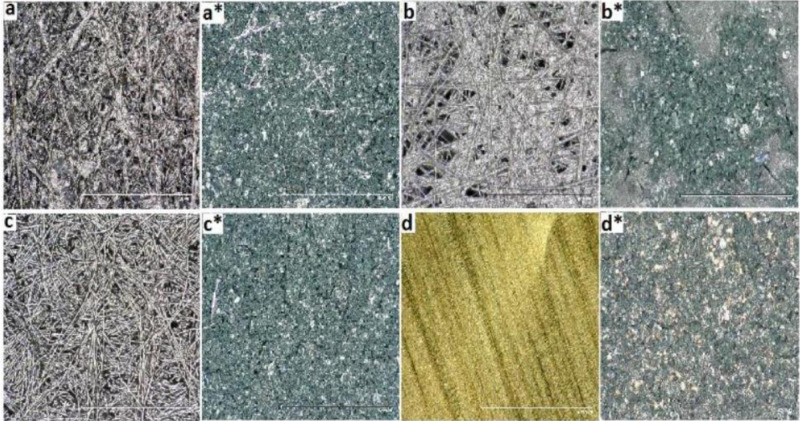
Microscopic images with 200 times magnification of (**a**) uncoated and (a*) coated active carbon paper (ACP)1, (**b**) uncoated and (b*) coated ACP2 (**c**) uncoated and (c*) coated ACP3 (**d**) uncoated and (d*) coated gold.

Different carbon papers, i.e., ACP1-3 as well as gold foil were punched out to small discs (8.0 mm dia.). They were washed in isopropanol to remove impurities and loose material and were dried at 60 °C under vacuum for at least 48 h. For gold electrodes, the surface was polished with diamond paste and rinsed with water and isopropanol and dried at 100 °C for 24 h. An amount of 20 mg of PANI-sample (S1, S2, S3) was suspended in 4 mL DMF and stirred thoroughly at room temperature, then placed in an ultrasonic sound bath for 30 min. The respective ACP’s and gold discs were coated with 200 μL of PANI-DMF suspension. Then the solvent was led to evaporate slowly at 25 °C for 12 h resulting in a thin and even layer of dry PANI on the composite. The coated PANI-composite material was dried under vacuum at 80 °C for 24 h to remove residual solvent. For pre-post comparison of the PANI-coating pictures were taken (Figure 2), the weight of each disc was determined (see Supporting Information Tables S4 and S5).

### 2.4. Manufacturing of the Supercapacitor Cell

In a battery cell casing two identical PANI-coated materials (8.0 mm dia.) were placed upon and below two glass fiber separators (2 × 0.26 mm thick, 10 mm dia., Whatman GF/A) with a platinum wire in between acting as a pseudo-reference electrode. As electrolyte 70 μL of 1.0 M aqueous H_2_SO_4_ solution flushed with Argon was used. The electrodes were connected to stainless steel plate current collectors (8.0 mm dia.) sealed in PEEK. Additionally, the platinum pseudo reference electrode was placed in a vessel filled with the same electrolyte (10 mL) and an Ag/AgCl reference electrode (sat. KCl in H_2_O) was used to measure the actual potential. The cell was placed on a cell stand with Peltier-heating/cooling unit. Before electrochemical measurements were done, there was a 45 min resting time to get a constant temperature of 25 °C.

## 3. Results and Discussion

### 3.1. Structural and Morphological Analysis

**Figure 3:**
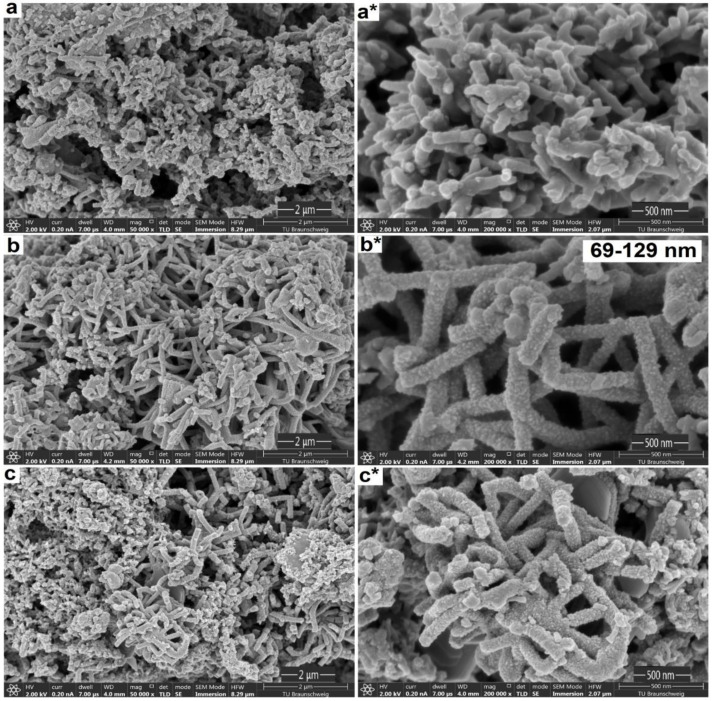
Surface morphology of (**a**–**c**) Polyaniline (PANI)-S1, S2 and S3 at (50 k times zoom); (a*–c*) PANI-S1, S2 and S3 at (200 k times zoom).

Figure 3 shows the different morphologies of the PANI composites (S1, S2 and S3) with variant sodium phytate dopant amounts. For PANI-S1 (0.5% (*w*/*v*) dopant, Figure 3a) irregular agglomerated short fibrous growth patterns were found. In contrast, for PANI-S2 (5% (*w*/*v*) dopant) long and uniform fiber interconnected structures with rough and porous morphology were observed (Figure 3b). The fibers had diameters of 69 nm to 129 nm and their structure revealed no random agglomerates as within the other samples. A further increase in the amount of dopant to 10% (*w*/*v*) for PANI-S3 led to thicker and shorter fibers with the occurrence of random aggregates (Figure 3c). This type of structure is not feasible for energy storage applications as the presence of side’s fibers with twists reduces the diffusion of electrolytes into the polymer matrix which in turn decreases the catalytic activity of the material [44,45]. In the case of PANI-S3, breakage of long fibers into small pieces is clearly seen with an increase in the diameter of fibers. This in turn reduces the electrical properties of the material [46]. 

An explanation for this dopant effect on the formation of fiber structures can cause a weak electrostatic repulsion between the polymer chains, which in PANI-S1 mainly leads to the formation of intergrown structures. In the case of PANI-S2, this electrostatic repulsion is high enough that a desirable morphology type is formed with a rough, highly porous, and fibrous nanostructure with pronounced connectivity and particle size. If the dopant is increased further, as in the case of PANI-S3, the repulsion can be too high, so that cleavage of the polymer chain is forced, and the structure is again distorted. The defined repulsion pattern gives PANI-S2 an interesting, desirable structure that is absent in other cases. Therefore, the multi-phosphate structure of sodium phytate provides a useful method for mass production of porous and fibrous nanostructures from PANI. 

It is known that such interconnected PANI nanofiber structures can be more advantageous for electrical and capacitive properties than wires and particles when they are used as electrode materials for supercapacitors. This can be attributed to large open channels of the pores with rough surfaces within the structures [47], since the fibrous and rough feature of PANI-S2 offers a large surface area which is favorable for the transport of electrons and ions and advantageous for good electrocatalysis properties [48,49,50].

The differences in morphology related directly to the surface area value, pore size distribution range and volume of the pores. The surface area of the PANI-S1, S2 and S3 samples was calculated by measuring nitrogen absorption and desorption isotherms using Brunauer–Emmett–Teller (BET) analysis (see Supporting Information, Figures S2–S4). PANI-S2 exhibited a significantly higher surface area value (230.5 m^2^g^−1^) than PANI-S1 (200.4 m^2^g^−1^) and PANI-S3 (101.5 m^2^g^−1^). Furthermore, the pore size distributions and volumes were measured by the Barrett–Joyner–Halenda (BJH) method. The pore size distribution of the samples is similar, ranging from 15.0–19.5 nm while the volume follows the same trend as with the surface area with 0.032 cm^3^g^−1^ for PANI-S1, 0.046 cm^3^g^−1^ for PANI-S2 and 0.011 cm^3^g^−1^ for PANI-S3, respectively. The results indicated that the reaction parameters of PANI-S2 with 5% sodium phytate dopant were most desirable to achieve fibrous and porous nanostructural networks with different connectivity, particle size and pore volume. It leads to enhanced ion transportation and accelerated electron transfer in the electrode material during the charge–discharge process, resulting in increasing the specific capacitance and consequently decreasing the electrical resistance [51,52,53].

To examine the elemental composition of PANI doped with sodium phytate, EDX analysis was used (see Supporting Information, Figure S5). The results show that all PANI samples contain C, O, Na, N and P. The detection of Na and P in all due samples the incorporation of the dopant illustrate the successful synthesis of PANI salts with effective assimilation of the dopant into the polymer backbone. The experimental results showed that increasing the dopant ratio increased the Na and P content in the salts up to 5%. A further increase in the dopant ratio reduces the content of the corresponding elements. The detected atomic ratios of sodium and phosphorus were 0.11% and 0.39% for PANI-S1, 1.05% and 1.69% for PANI-S2 and 0.89% and 0.97% for PANI-S3. The EDX results apparently confirmed that all samples were doped successfully, in which PANI-S2 has the highest percentage of Na and P. In particular, the high phosphorus content of the PANI-S2 sample is worthwhile because it reduces the agglomeration of the polymer chains and the barrier height, creates a deep interaction for the intra- and intermolecular charge delocalization and is intended to improve the electrical conductivity [54].

### 3.2. Electrochemical Analysis

To confirm that the PANI samples have the potential as electrode material for supercapacitors, the electrochemical properties were investigated by cyclic voltammetry (CV), galvanostatic charge–discharge (GCD), and electrochemical impedance spectroscopy (EIS). The PANI samples were coated on three different active carbon papers with fibrous webbed structure (ACP1) and microporous surface layer (ACP2 and ACP3) as well as on gold foil for better comparison with literature. As for supercapacitors a high specific surface area is a key feature for great performance, the ACP@PANI composites should favor stronger pseudocapacitance [55,56].

**Figure 4:**
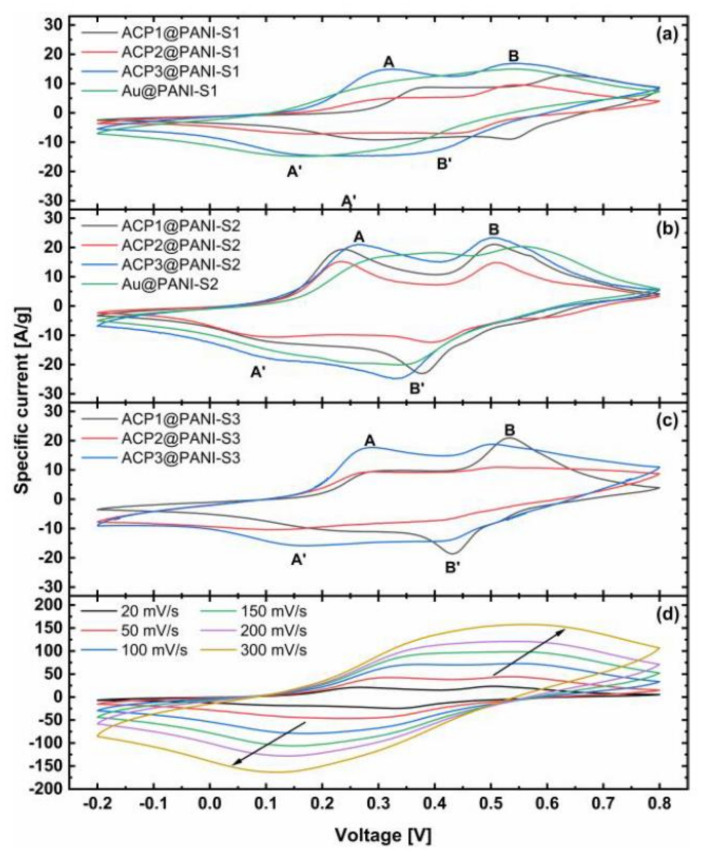
Cyclic voltammograms of the different composites (ACP1-3) coated with (**a**) PANI-S1, (**b**) PANI-S2 and (**c**) PANI-S3 with a scan rate of 20 mVs^−1^ at 25 °C. (**d**) ACP3@PANI-S2 at altering scan rates.

Since the cyclic voltammetry of PANI derivatives has been extensively described in literature before, they are only briefly presented here. Figure 4 shows the cyclic voltammograms of the ACP@PANI- and Au@PANI over a potential window of 1 V between −0.2 V and 0.8 V (vs. Ag/AgCl, sat. KCl in H_2_O), with a scan rate of 20 mVs^−1^ and ACP3@PANI-S2 with altering scan rates. All composites exhibit a nearly rectangular shape with two well-resolved redox pairs. The shape is characteristic for supercapacitors made of PANI. Each slope can be partitioned in three areas: (1) below 0.2 V corresponds to the main region of the insulation state of leucomeraldine, (2) between 0.3 V to 0.4 V occurs the first redox pair A/A’, which relates to the half oxidized, conducting emeraldine form and (3) above 0.4 V to 0.5 V the second redox reaction B/B’ takes place to the complete oxidized pernigraniline. These redox pairs embedded in a high background current indicate pseudocapacitive performance of PANI. The anodic and cathodic peaks were observed to be symmetric, reflecting superior reversibility of the relevant redox reactions and most of the energy is stored by Faradic reactions [57]. CV’s at different scan rates, e.g., ACP3@PANI-S2, show a shift toward scan direction with increasing scan rates and increasing specific currents resulting in a merging of the two separated redox pairs. The peak currents are linearly proportional to the scan rate indicating that the reaction kinetics become surface limited, but therefore exhibit high rate capability [58,59].

The results show that the combination of ACP3@PANI has an improved specific capacitance (120–180 Fg^−1^ more) as compared with the other samples. It can be anticipated that the hydrophilic microporous structure of ACP3 with its much larger surface area benefits the electrochemically active surface of the composites. This synergizes well with the overall better properties of PANI-S2 leading to an excellent specific capacitance of 1102.2 ± 1.8 Fg^−1^. In contrast to them the ACP1- and ACP2@PANI composites exhibit significantly lower specific capacitances probably based on their higher hydrophobic treatment with PTFE-binder, which insulates the carbon fibers and hinders the effective diffusion into the pores. CV experiments of the bare APCs also showed that the supporting carbon papers made no contribution to capacitances because their specific capacities were four orders of magnitude lower than for those of the respective ACP@PANI composites (see Supporting Information, Figure S1). Additionally, the Au@PANI samples exhibit higher specific capacitances than the ACP1- and ACP2@PANI composites, albeit the gold surface has a hydrophobic character. The difference is that the gold discs merely act as PANI carriers unable to provide a porous structure. This illustrates perfectly that conducting polymers form much more complicated morphologies than, e.g., metal oxides, intermetallic composites, or MOF’s. The length and the dispersity of the PANI strains and their either more or less intense interaction between each other and with the functional groups on the surface of the ACP’s can lead to disordered diffusion networks that can affect the reaction rate capability tremendously [55].

**Figure 5:**
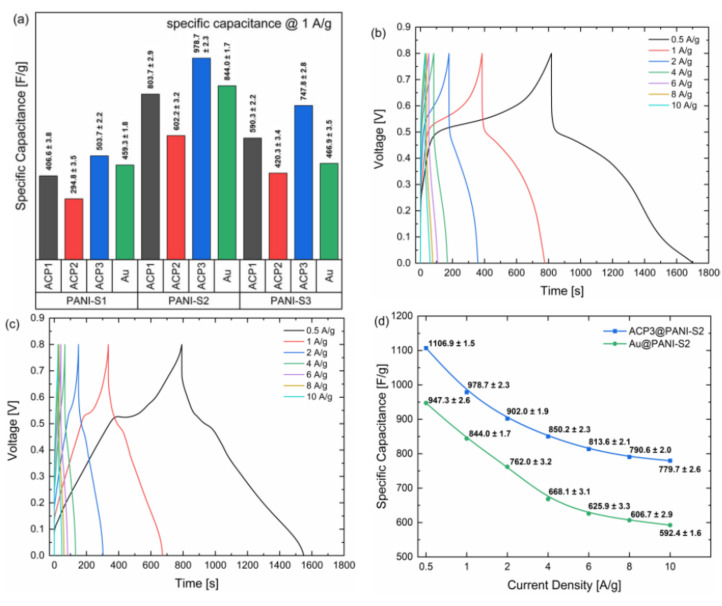
(**a**) Specific capacitances of the different PANI composites at a current density of 1 A g^−1^; (**b**) Galvanostatic charges-discharge curves of ACP3@PANI-S2 at different current densities; (**c**) Galvanostatic charge-discharge curves of Au@PANI-S2 at different current densities; (**d**) Variation of current densities and their respective capacitances of ACP3@PANI-S2 and Au@PANI-S2.

To exactly reveal the electrochemical capacitive performance, galvanostatic charge–discharge experiments were made at a current density of 1 Ag^−1^ (Figure 5a). Specific capacitances were calculated from the respective discharge curves of the composites in a potential window of 0–0.8 V (vs. Ag/AgCl, sat. KCl in H_2_O). They correlate well with the CV results. The specific capacitances of ACP3@PANI-S2 and Au@PANI-S2 with 978.7 ± 2.3 Fg^−1^ and 844.0 ± 1.7 Fg^−1^, respectively, are the highest among all other samples, since they showed the best performance. This admirable specific capacitance of ACP3@PANI-S2 is much superior to reported PANI-carbon based materials in three-electrode setups (Table 1).

Figure 5b,c show the respective GCD results for ACP3@PANI-S2 and Au@PANI-S2 at different current densities between 0.5 Ag^−1^ and 10 Ag^−1^. The GCD curves of ACP3@PANI-S2 show a typical discharge behavior for PANI coated on active carbon paper with a relatively large IR-drop of roughly 0.3 V due to a large equivalent series resistance (ESR) and broadened and bulky discharge slopes (Figure 5b). The charging slopes show a fast increase in voltage from 0 V to 0.3 V likewise due to the ESR followed by a steep slope of the curve which flattens at about 0.5 V to 0.65 V, because of the oxidation of emeraldine to pernigraniline, which is the major redox transition where energy is stored in. In contrast, Au@PANI-S2 exhibits a distorted triangular shape with a shoulder at 0.5 V (Figure 5c). The IR-drop is much smaller than for the ACP composites with 0.1 V probably due to the much lower ohmic resistance of the metal. The triangular shape of Au@PANI-S2 is characteristic for an ideal capacitance. The distortion at 0.5 V is caused by the pseudocapacitive behavior due to the fast reaction of the PANI redox processes, as with ACP3@PANI-S2. However, the charging and discharging time constants are significantly lower for Au@PANI-S2, indicating a lower contribution of PANI-S2 to the charge storage process. This is likely due to the porous structure of the ACP, which allows the electrolyte ions to penetrate the internal structure of the PANI much better. In addition, it can be observed that the respective charge–discharge time constants for Au@PANI-S2 decrease by 33-fold and 31-fold, respectively, when the current density increases from 0.5 Ag-1 to 10 Ag-1 by a factor of 20, while for ACP3@PANI-S2 it is 28-fold for both processes. This shows clearly that the charging processes of Au@PANI-S2 are more sensitive to increases in current density than for ACP3@PANI-S2. However, ACP3@PANI-S2 and Au@PANI-S2 exhibit similar high coulombic efficiencies of 92% and 98% at 0.5 Ag^−1^, respectively. At a current density of 10 Ag^−1^, only a slight decrease in the coulomb efficiencies was observed (89% for ACP3 @ PANI-S2, 91% for Au @ PANI-S2), which are mainly due to an increase in the IR drop. This clearly shows that different current densities have a negative non-linear influence on the charging and discharging times of the composites. The efficiency at a stable current density of the processes, on the other hand, remains almost constant over the selected range. These effects are also reflected in the capacity measurements and capacity retention capability. 

Figure 5d presents the comparative results of the specific gravimetric capacitance of the individual ACP3@PANI-S2 and Au@PANI-S2 at different current densities. The overall specific capacitance retentions for the two composites are 70% and 63%, respectively. Between 1 Ag^−1^ and 6 Ag^−1,^ Au@PANI-S2 showed an especially stronger decrease in specific capacitance than ACP3@PANI-S2 with a retention of 74% to 83%, while at the higher current densities the values become more stable (both 95–96% retention). Those results revealed that the ACP3@PANI-S2 composite has better sustainability to high current and rate performance compared with Au@PANI-S2 composite. 

**Figure 6:**
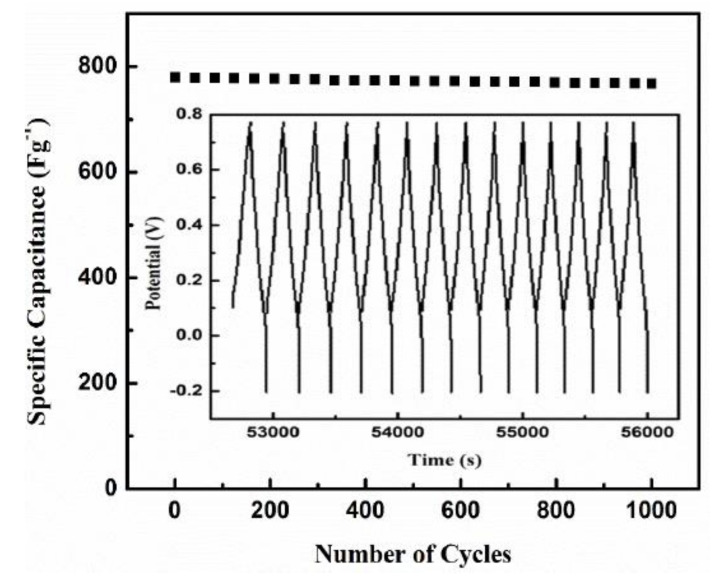
Cyclic stability of ACP3@PANI-S2 composite symmetric supercapacitor at 10 Ag^−1^ for 1000 cycles.

Cycling stability is a key factor for operational supercapacitors. In particular, supercapacitors based on conducting polymers often experience limited cyclic stability because of the shrinking and swelling of the polymers during its charging–discharging operation [55]. The cycling performance of ACP3@PANI-S2 at a high current density of 10 Ag^−1^ is shown in Figure 6.

The composite exhibits excellent capacitance retention of over 96% after 1000 cycles. According to literature, this capacitance retention is superior to that of many other supercapacitors based on PANI (typically 60∼85% retention for 1000 cycles) [67,68]. The achievement of high cycling performance for our electrode material could be accredited to the interconnected, porous nanostructure morphology that prevents the shrinking and swelling problems of the polymer network during the intensive cycling process.

**Figure 7:**
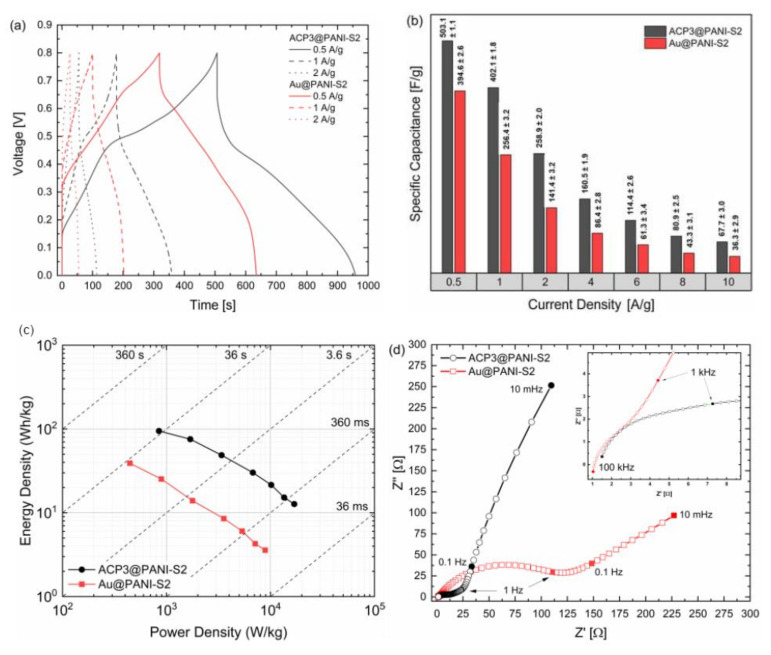
(**a**) Two-electrode galvanostatic charge–discharge of ACP3- and Au@PANI-S2 at different current densities; (**b**) Variation of current densities and their respective capacitances of ACP3@PANI-S2 and Au@PANI-S2 in two-electrode cell setup; (**c**) Ragone plot of ACP3@PANI-S2 and Au@PANI-S2; (**d**) Nyquist plots of ACP3@PANI-S2 and Au@PANI-S2 using a perturbation amplitude of 10 mV at open circuit potential.

Since commercial supercapacitors are produced and utilized in a two-electrode cell setup, we decided to investigate the performance of the ACP3@PANI-S2 and Au@PANI-S2 under similar conditions. It is known that two- and three-electrode cell setups differ significantly from each other and that the actual performance with two electrodes is usually half or less in terms of capacitance [69]. For that reason, we manufactured the same supercapacitor cells, but without reference electrode and performed GCD and EIS experiments (Figure 7).

In Figure 7a the galvanostatic charge–discharge curves of ACP3@PANI-S2 and Au@PANI-S2 are shown. They became more triangular-shaped than in the three-electrode cell setup and charge and discharge times reduced by half or more. However, some characteristics remained the same, such as the larger IR-drop of the ACP3@PANI-S2 composite and its much higher specific capacitance compared to the Au@PANI-S2 composite (Figure 7b). Noteworthy is that the specific capacitances decrease significantly more with increasing current densities than in the three-electrode setup. The specific capacitance retention of ACP3@PANI-S2 is about 80% for 0.5 Ag^−1^ to 1 Ag^−1^. With increasing current densities between 2 Ag^−1^ and 6 Ag^−1^ the retention decreases to 62–64% and stabilizes for higher current densities values between 70–83%. In contrast, the specific capacitance retention for Au@PANI-S2 is rather stable between 0.5 Ag^−1^ and 8 Ag^−1^ with 61–71% and possesses the same retention value as ACP3@PANI-S2 at 10 Ag^−1^ with 83%. Differences in capacitance retention might come from changes in ion transport inside the composite and charge transfer processes, due to equal polarization of working and counter electrode in the two-electrode setup. These changes influence the ACP3@PANI-S2 composite much more since its surface area and pore volume is larger. Therefore, the specific capacitance of ACP3@PANI-S2 in a two-electrode cell setup is much higher than reported PANI-carbon based materials (Table 2).

Figure 7c represents the Ragone plots of the two PANI-S2 composites. The energy density can approach 95 Wh kg^−1^ at a power of 846 W kg^−1^ for ACP3@PANI-S2. At a high-power density of 16.9 kW kg^−1^, the energy density can still be kept at 13 Wh kg^−1^. For Au@PANI-S2, energy density is a third and power density roughly half of the respective value for ACP3@PANI-S2. Obtaining a high-power density, while possessing a relatively large energy density, further proves that the PANI-S2 nanocomposite possesses an enhanced electrochemical performance as electrode material. Therefore, it can be used for any application where high energy and power is required.

EIS was used to further study the internal resistance, charge transfer kinetics and ion diffusion processes of the ACP3- and Au@PANI-S2 composites (Figure 7d). The frequency range was between 100 kHz to 10 mHz using a perturbation amplitude of 10 mV. The ESR values of ACP3@PANI-S2 and Au@PANI-S2 obtained from the intercept of the plot on the real axis (Z’) at high frequency were 1.48 Ω and 1.03 Ω, respectively, reflecting a very good ionic conductivity of the supercapacitors and low internal resistance of the electrodes. In the middle frequency range up to 1 Hz with one semicircle the processes were assigned to charge–transfer reactions of the polymer. The charge transfer resistance of ACP3@PANI-S2 had a much smaller R_ct_ (19 Ω) than that of Au@PANI-S2 (114 Ω) which can be attributed to a shorter ion diffusion pathway at the electrode/electrolyte interface [73]. The low R_ct_ value proved that the ACP3 composite supports the electrolyte ions to penetrate the polymer and access the inner layer of the nanosized polymer matrix. At a low-frequency range, both composites exhibit a monotonically increasing line, which was attributed to ion diffusion. For ACP3@PANI-S2, the slope of the straight line was more distinct toward the imaginary impedance axis (Z”) and is related to a more ideal polarizable capacitance [74]. This proves that ACP3@PANI-S2 is the better electrode material for supercapacitor application, due to an excellent ion conductivity, low internal resistance, and good charge transfer performance.

## 4. Conclusions

Sodium phytate doped 3D PANI nanofibers anchored carbon paper electrodes can effectively be combined into symmetric supercapacitor assembly to fabricate light, high-performance supercapacitors. A test series with different dopant amounts was made, showing that the optimum of 5% dopant during synthesis favors the formation of long and uniform nanofiber (69–129 nm) interconnected structures with exceptionally large surface area (230.5 m^2^g^−1^) and pore volume (0.046 cm^3^g^−1^). Overall, the supercapacitors exhibited particularly good performance, rate capability and cycle stability. It is noteworthy that the combination of PANI nanofibers and carbon paper with its microporous surface morphology showed a synergetic effect, which led to a superior specific capacity of 1106.9 ± 1.5 Fg^−1^ and 779 ± 2.6 Fg^−1^ at a current density of 0.5 Ag^−1^ and 10 Ag^−1^, respectively. The symmetrical supercapacitor cell setup showed high capacity retention of 96% at 10 Ag^−1^ for 1000 charge–discharge cycles with very low internal resistance (1.48 Ω) and charge transfer resistance (19 Ω). Moreover, the gravimetric energy density of the device was 95 Wh kg^−1^ at a power of 846 W kg^−1^. At a high-power density of 16.9 kW kg^−1^, the energy density still remained 13 Wh kg^−1^. With respect to practical applications, we believe that the presented methods provide an efficient and promising way to produce electrode material with low cost, no environmental hazards, easy processability and excellent energy-storage performance.

## Figures and Tables

**Table 1:** Comparison of the Specific Capacitance of PANI-carbon-based materials in three-electrode system.

Materials	Current Density ^a^	C_sp_	Year	Reference
PANI-carbon microspheres	1.0 Ag^−1^	500 Fg^−1^	2018	[60]
PANI-biomas porous carbon	1.0 Ag^−1^	402 Fg^−1^	2018	[61]
Activated carbon PANI	0.2 Ag^−1^	465 Fg^−1^	2018	[62]
PANI-cellulose derived porous carbon	1.0 Ag^−1^	765 Fg^−1^	2019	[63]
Activated carbon PANI	1.0 Ag^−1^	520 Fg^−1^	2019	[64]
PANI-carbon cloth	25 mAcm^−2^	438 Fg^−1^	2019	[65]
PANI-GO nano-composite	10 Ag^−1^	658 Fg^−1^	2020	[66]
ACP3-coated sodium phytate doped PANI-S2	1.0 Ag^−1^ 10.0 Ag^−1^	978.7 ± 2.3 Fg^−1^ 779.7 ± 2.6 Fg^−1^	Present work	

^a^ Electrolyte in all cases 1 M H_2_SO_4_.

**Table 2:** Comparison of the Specific Capacitance of PANI based materials in two-electrode system.

Materials	Current Density ^a^	C_sp_	Year	Reference
Commercial carbon paper coated PANI	0.1 Ag^−1^	174 Fg^−1^	2016	[70]
PANI carbon cloth	1.0 Ag^−1^	68 Fg^−1^	2016	[71]
PANI activated carbon nanocomposite	1.0 Ag^−1^	142 Fg^−1^	2019	[72]
Activated carbon-PANI	1.0 Ag^−1^	520 Fg^−1^	2019	[64]
PANI carbon cloth	25 mAcm^−2^	247 Fg^−1^	2019	[65]
PANI-GO nano-composite	1.0 Ag^−1^	264 Fg^−1^	2020	[66]
ACP-coated sodium phytate doped PANI	0.5 Ag^−1^ 1.0 Ag^−1^	503.1 ± 2.4 Fg^−1^ 402.1 ± 3.3 Fg^−1^	Present work	

^a^ Electrolyte in all cases 1 M H_2_SO_4_.

## Supplementary Materials

The following are available online at https://www.mdpi.com/2073-4360/12/11/2705/s1, Tables S1–S3: Material information for the carbon electrodes ACP1-3, Table S4,S5: Weight of the active PANI material for CV, EIS and GCD, Figure S1: CV of the support material without PANI, Figures S2–S4: BET and BJH experiments of PANI-S1, S2, and S3, Figure S5: EDX analysis.
