# Supplementary material for: 3D Polyaniline Nanofibers Anchored on Carbon Paper for High-Performance and Light-Weight Supercapacitors
Source: Polymers (Basel). 2020 Nov 16;12(11):2705. doi: 10.3390/polym12112705 (PMC7696344; doi:10.3390/polym12112705)
Supplement: Supplementary file 1 [file polymers-12-02705-s001.pdf]

polymers

MDPI

Supporting Information

3D Polyaniline nanofibers anchored on carbon paper for high performance and light weight supercapacitors

Sami ur Rahman [1], Philipp Röse [2], Mit Surati[2], Anwar ul Haq Ali Shah [3], Ulrike Krewer [2*] and Salma Bilal [1*],

[1] National Centre of Excellence in Physical Chemistry 1, University of Peshawar, 25120 Peshawar, Pakistan; samiurrahman720@gmail.com (S.R)

[2] Karlsruhe Institute of Technology (KIT), Institute for Applied Materials, 76131 Karlsruhe, Germany; philipp.roese@kit.edu (P.R.); mitsurati@gmail.com (M.S)

[3] Institute of Chemical Science, University of Peshawar, 25120 Peshawar, Pakistan; anwarulhaqalishah@uop.edu.pk (A.A.S)

* Correspondence: salmabilal@uop.edu.pk; Tel.: 0092-919216766 (S.B.); ulrike.krewer@kit.edu; Tel: +49 721 608-47490 (U.K)

Content:

**Material information: Table S1-S3**	**S. 2**
**PANI loading: Table S4-S5**	**S. 3**
**Cyclic voltammogram of the support: Figure materials without PANI: Figure S1**	**S. 4**
**BET and BJH Data: Figure S2-S4**	**S. 5**
**EDX Data: Figure S5**	**S. 7**

Table S1. Material information for the Sigracet 29AA (ACP1) carbon electrode.

Material Type	Carbon Fiber Paper
Thickness	190 microns +/- 30 microns
Basic Weight (g/m²)	40 +/- 10 g/m²
Porosity (%)	80%
Compressibility	ca. 34% (at 1 MPa)
Electrical Resistivity (through plane)	< 5 mΩcm² (milliohms cm²)
IP Pressure Drop	0.3 bar (at 1 MPa)
PTFE Treatment	PTFE Treatment Applied
Microporous Layer	No Microporous Layer Applied

Table S2. Material information for the Sigracet 35BC (ACP2) carbon electrode.

Material Type	Carbon Fiber Paper
Thickness	325 microns +/- 25 microns
Basic Weight (g/m²)	110 +/- 10 g/m²
Air Permeability (s)	1.50 +/- 1.00 cm³/(cm²*s)
Porosity (%)	80%
Electrical Resistivity (through plane)	< 15 mΩcm² (milliohms cm²)
PTFE Treatment	5%
Microporous Layer	Yes, on one side
Material Type	Carbon Fiber Paper

Table S3. Material information for the Freudenberg H23C2 (ACP3) carbon electrode.

Material Type	Carbon Fiber Paper
Thickness	255 microns
Basic Weight (g/m²)	135 +/- 10 g/m²
Porosity (%)	80%
Compressibility	ca. 16% (at 1 MPa)
Electrical Resistivity (through plane)	< 10 mΩcm² (milliohms cm²)
Decomposition Temperature	350 °C
PTFE Treatment	40%
Microporous Layer	Yes, on one side (the coated side)

Table S4. Weight of the active PANI material for CV and EIS experiments after coating.

Electrode	PANI sample	Weight active Material / mg
ACP1	S1	0,510
	S2	0,260
	S3	0,210
ACP2	S1	0,137
	S2	0,340
	S3	0,166
ACP3	S1	0,128
	S2	0,078
	S3	0,012
Au	S1	0,207
	S2	0,522
	S3	0,037

Table S5. Weight of the active PANI material for GCD experiments after coating.

Electrode	PANI sample	Weight active Material / mg
ACP1	S1	2,255
	S2	0,520
	S3	0,815
ACP2	S1	0,375
	S2	1,000
	S3	1,410
ACP3	S1	1,000
	S2	0,660
	S3	0,255
Au	S1	2,185
	S2	2,325
	S3	3,735

Cyclic voltammetry of the support materials

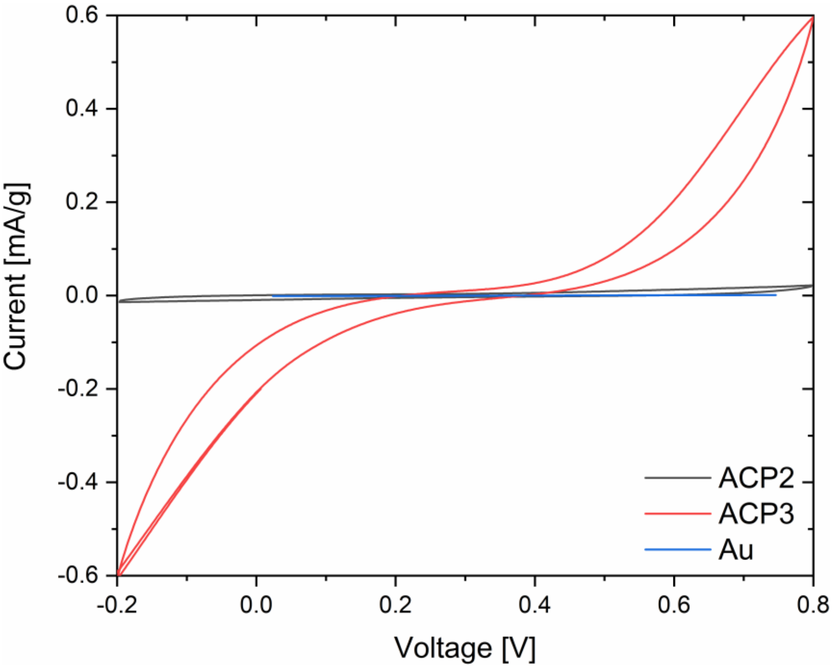

Figure S1: CV of APC2, APC3 and Au in 1M H2SO4 with PANI at a scan rate of 100 mVs^{-1}.

Results of the BET and BJH-Experiments

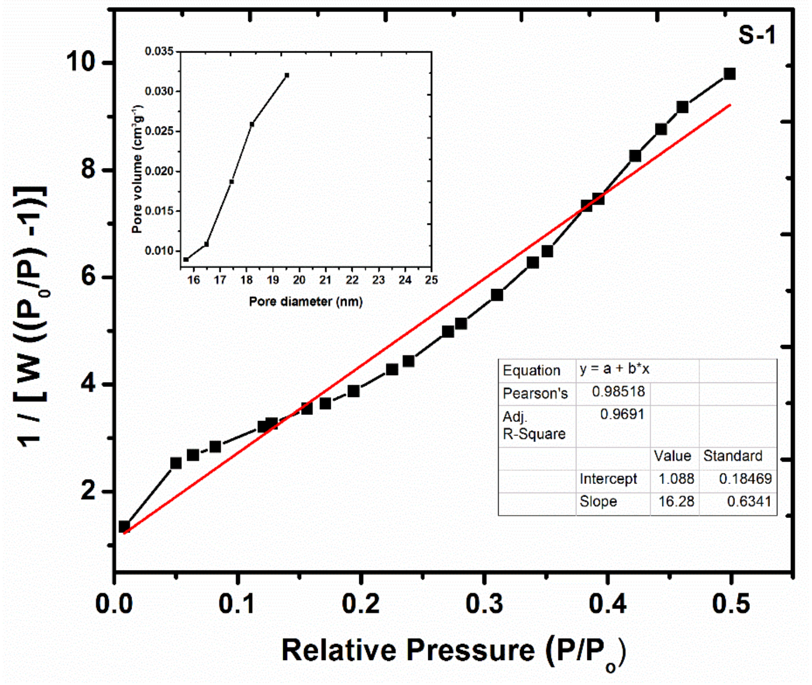

Figure S2. Nitrogen adsorption Curve of PANI-S1 while the inset curve shows pore size distribution and pore volume.

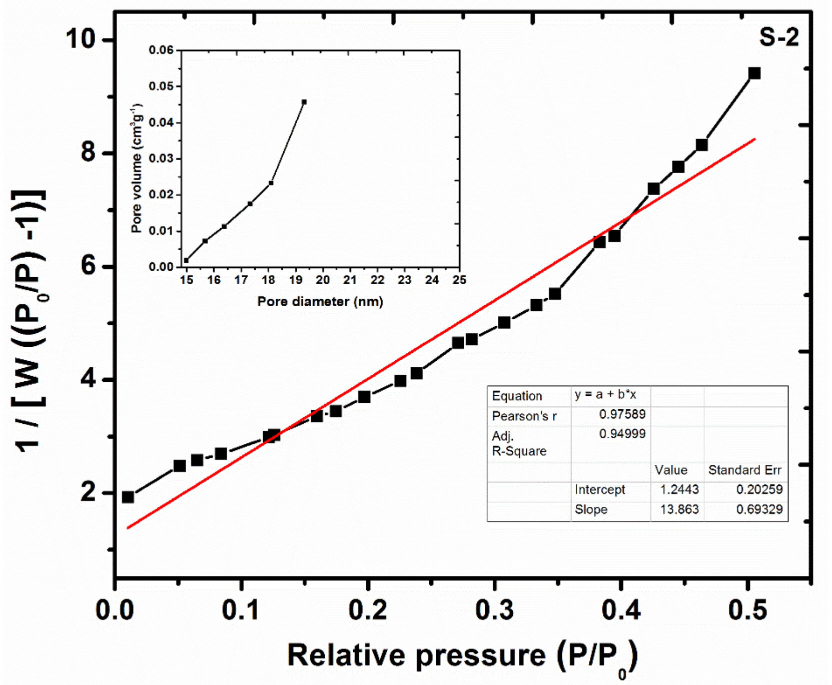

Figure S3. Nitrogen adsorption Curve of PANI-S2 while the inset curve shows pore size distribution and pore volume.

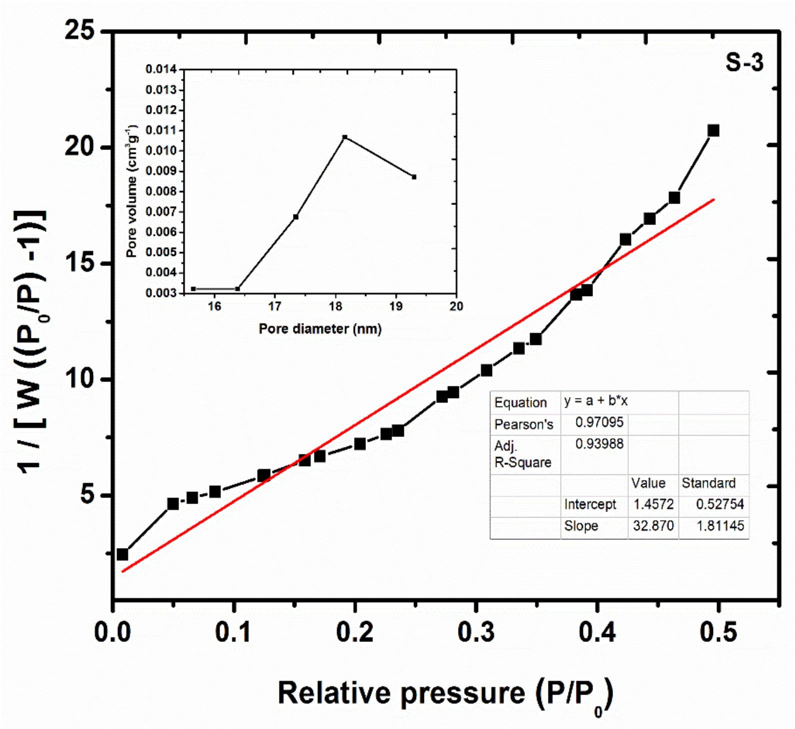

Figure S4. Nitrogen adsorption Curve of PANI-S3 while the inset curve shows pore size distribution and pore volume.

EDX Analysis

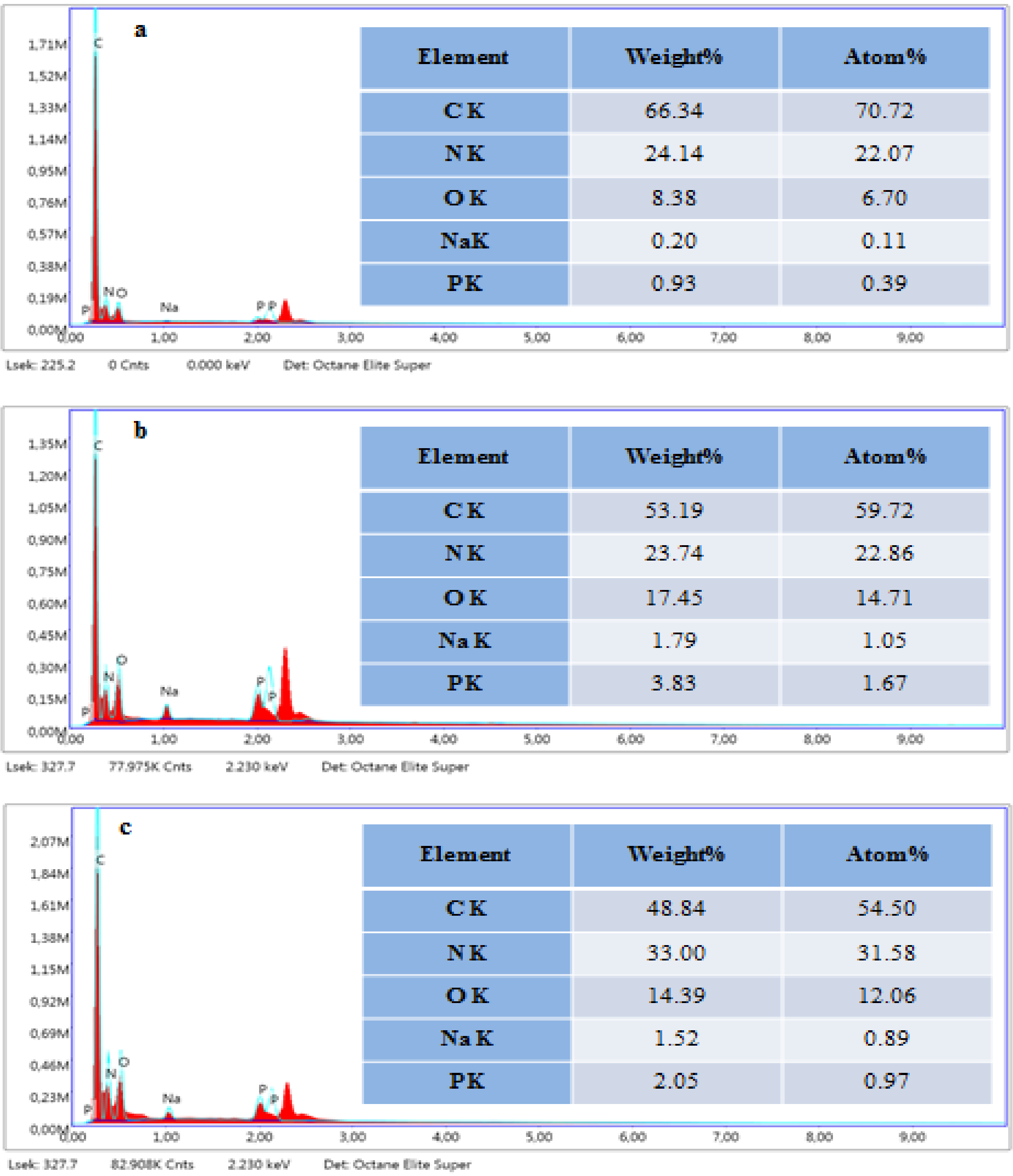

(a)

Element	Weight%	Atom%
C K	66.34	70.72
N K	24.14	22.07
O K	8.38	6.70
NaK	0.20	0.11
PK	0.93	0.39

(b)

Element	Weight%	Atom%
C K	53.19	59.72
N K	23.74	22.86
O K	17.45	14.71
Na K	1.79	1.05
PK	3.83	1.67

(c)

Element	Weight%	Atom%
C K	48.84	54.50
N K	33.00	31.58
O K	14.39	12.06
Na K	1.52	0.89
PK	2.05	0.97

Figure S5: EDX analysis of PANI-S1, S2 and S3.

© 2020 by the authors. Submitted for possible open access publication under the terms and conditions of the Creative Commons Attribution (CC BY) license (http://creativecommons.org/licenses/by/4.0/).
